# Design of the DISCovery project: tailored work-oriented interventions to improve employee health, well-being, and performance-related outcomes in hospital care

**DOI:** 10.1186/1472-6963-13-66

**Published:** 2013-02-19

**Authors:** Irene MW Niks, Jan de Jonge, Josette MP Gevers, Irene LD Houtman

**Affiliations:** 1 Department of Industrial Engineering & Innovation Sciences, Human Performance Management Group, Eindhoven University of Technology, PO Box 513, 5600 MB, Eindhoven, The Netherlands; 2 Quality and Safety Department, Rijnstate Hospital, PO Box 9555, 6800 TA, Arnhem, The Netherlands; 3 TNO, Work and Health, Polarisavenue 151, 2132 JJ, Hoofddorp, The Netherlands

**Keywords:** Intervention study, Employee health, Performance, Hospital care

## Abstract

**Background:**

It is well-known that health care workers in today’s general hospitals have to deal with high levels of job demands, which could have negative effects on their health, well-being, and job performance. A way to reduce job-related stress reactions and to optimize positive work-related outcomes is to raise the level of specific job resources and opportunities to recover from work. However, the question remains how to translate the optimization of the balance between job demands, job resources, and recovery opportunities into effective workplace interventions. The aim of the DISCovery project is to develop and implement tailored work-oriented interventions to improve health, well-being, and performance of health care personnel.

**Methods/Design:**

A quasi-experimental field study with a non-equivalent control group pretest-posttest design will be conducted in a top general hospital. Four existing organizational departments will provide both an intervention and a comparison group. Two types of research methods are used: (1) a longitudinal web-based survey study, and (2) a longitudinal daily diary study. After base-line measures of both methods, existing and yet to be developed interventions will be implemented within the experimental groups. Follow-up measurements will be taken one and two years after the base-line measures to analyze short-term and long-term effects of the interventions. Additionally, a process evaluation and a cost-effectiveness analysis will be carried out.

**Discussion:**

The DISCovery project fulfills a strong need for theory-driven and scientifically well-performed research on job stress and performance interventions. It will provide insight into (1) how a balance between job demands, job resources, and recovery from work can be optimized, (2) the short-term and long-term effects of tailored work-oriented effects, and (3) indicators for successful or unsuccessful implementation of interventions.

## Background

Hospitals need to work more efficient than ever before to increase the quality of health care and at the same time reduce costs, which places a higher burden on health care staff. As a consequence, health care workers are often imposed with highly demanding cognitive, emotional, and physical work tasks [1-3]. Such demanding tasks that require effort are also referred to as job demands [4,5]. High levels of job demands can have negative effects on employees’ health, well-being, and job performance [6], unless workers have sufficient job resources to cope with their demanding jobs [7]. Job resources can be described as work-related assets (i.e., tools, information, people, opportunities) that can be employed to deal with job demands [4]. Examples of job resources are workplace social support and job autonomy. Because job demands often cannot be reduced, the idea to increase job resources instead to combat strain is appealing for today’s working life in health care.

A new theoretical model regarding the stress-buffering role of job resources, is the Demand-Induced Strain Compensation (DISC) Model [8,9]. In addition to other job stress models, the theoretical basis of the DISC Model is premised on self-regulatory processes of match at work. The DISC Model assumes that job demands, job resources, and job-related outcomes are multidimensional factors comprised of cognitive, emotional, and physical components. It proposes employees to activate functional, corresponding job resources, to mitigate the negative effects of high job demands. In other words, different kinds of high job demands (i.e., cognitive, emotional, or physical) can best be compensated for by corresponding kinds of job resources to counteract negative job-related outcomes. For instance, emotional support from colleagues may particularly help to reduce emotional exhaustion caused by emotional labour (e.g., aggressive patients). Research findings have indeed shown that moderating effects are found more often for matching resources than for non-matching resources [4,10]. Furthermore, it is proposed that optimal conditions for active learning, growth, creativity, and performance exist when a balanced mixture of (high) demands and corresponding job resources occurs [8]. Employees need both challenging demands and usable, matching, job resources to learn and to grow, and to feel well. Indeed, a number of DISC studies showed that the combination of high cognitive demands and high cognitive resources was associated with different forms of cognitive well-being, such as high active learning and creativity [11], and professional efficacy [12].

Equally important as the role of (matching) job resources, is the process of recovering from job demands [13]. Recovery is defined as the process opposite to the strain process, that enables employees to regain the energy they expended at work and to rebuild resources that have been depleted during work [14]. Recently, recovery from work was added to the DISC Model, here also distinguishing a cognitive component (e.g., no longer thinking of work), an emotional component (e.g., putting all emotions from work aside), and a physical component (e.g., shaking off physical exertion) [15]. This is in line with Sonnentag and Niessen [16], who proposed that a full degree of off-job recovery is attained when the employee feels that cognitive and physical as well as emotional systems called upon during work have returned to their baseline levels. According to De Jonge et al. [15], recovery that matches particular demands will be most effective (e.g., emotional recovery in relation to emotional demands). The idea is that matching recovery may foster health, by restoring the specific internal resources that have been depleted by specific job demands. Overall, the expanded DISC Model predicts that both job resources and recovery from work that correspond with the specific job demands will most effectively counteract negative effects of job demands, and create optimal conditions for health and performance. For example, high emotional job demands can lead to strong feelings of emotional exhaustion, unless employees have high emotional resources as well as a high level of emotional recovery from work.

### Study objectives

As there is a gap between theoretical knowledge gained from work stress and performance models and their practical implications [17], this study will apply key propositions of the expanded DISC Model to real practice. The main purpose of the DISCovery project is to develop and implement tailored work-oriented interventions to improve a healthy working life and job performance in a general hospital. Health care workers are ideally suited for practical applications of the DISC Model, because all three types of job demands (i.e. heavy physical work, negative emotion work, and complex work under pressure) are present in their work.

In line with the DISC model, the core question is how different types (i.e., cognitive, emotional, or physical) of job demands, job resources, and recovery during and after working hours can be optimized to improve health and performance of health care workers. We expect that interventions targeted primarily at work, i.e., at specific job demands and particularly at changing corresponding resources and recovery aspects, will reduce detrimental effects and enhance beneficial effects. In other words, by providing employees with necessary, matching job resources and recovery opportunities for coping with job demands, hospitals may prevent unnecessary stress and strain, and improve worker well-being and job performance. Thereby, the study can contribute to human resources strategies to keep current staff in the house and to ensure longer employment. Another contribution of this study will be to the area of patient safety and medical treatment errors. Although critical issues in this field have received a great deal of attention lately, little is known about the effects of job demands, job resources, and recovery from work on patient safety and treatment errors [18]. Following DISC theory, it is expected that a well-balanced match of job demands, job resources, and recovery will lead to fewer treatment errors and better patient safety, due to increased job performance and reduced stress reactions (e.g., less concentration problems). Therefore, intervention effects are also assessed in terms of safety and error outcomes. The study should shed light on what the short-term (e.g., health, motivation, optimal resourcing, and recovery) and long-term (e.g., safety, performance, absenteeism, and turnover) effects of the intervention program are. Finally, since there is a strong need for research exploring the processes that influence intervention outcomes [19], special attention will be paid to learn why and under what circumstances work-oriented interventions succeed. Indicators for successful or unsuccessful implementation of interventions will be investigated, such as supervisor involvement and employee attitude towards the interventions. The implementation goal following this project includes the substantial involvement of stakeholders, as well as the dissemination and embeddedness of findings of the study in health care practice.

## Methods and design

### Study design

**Figure 1:**
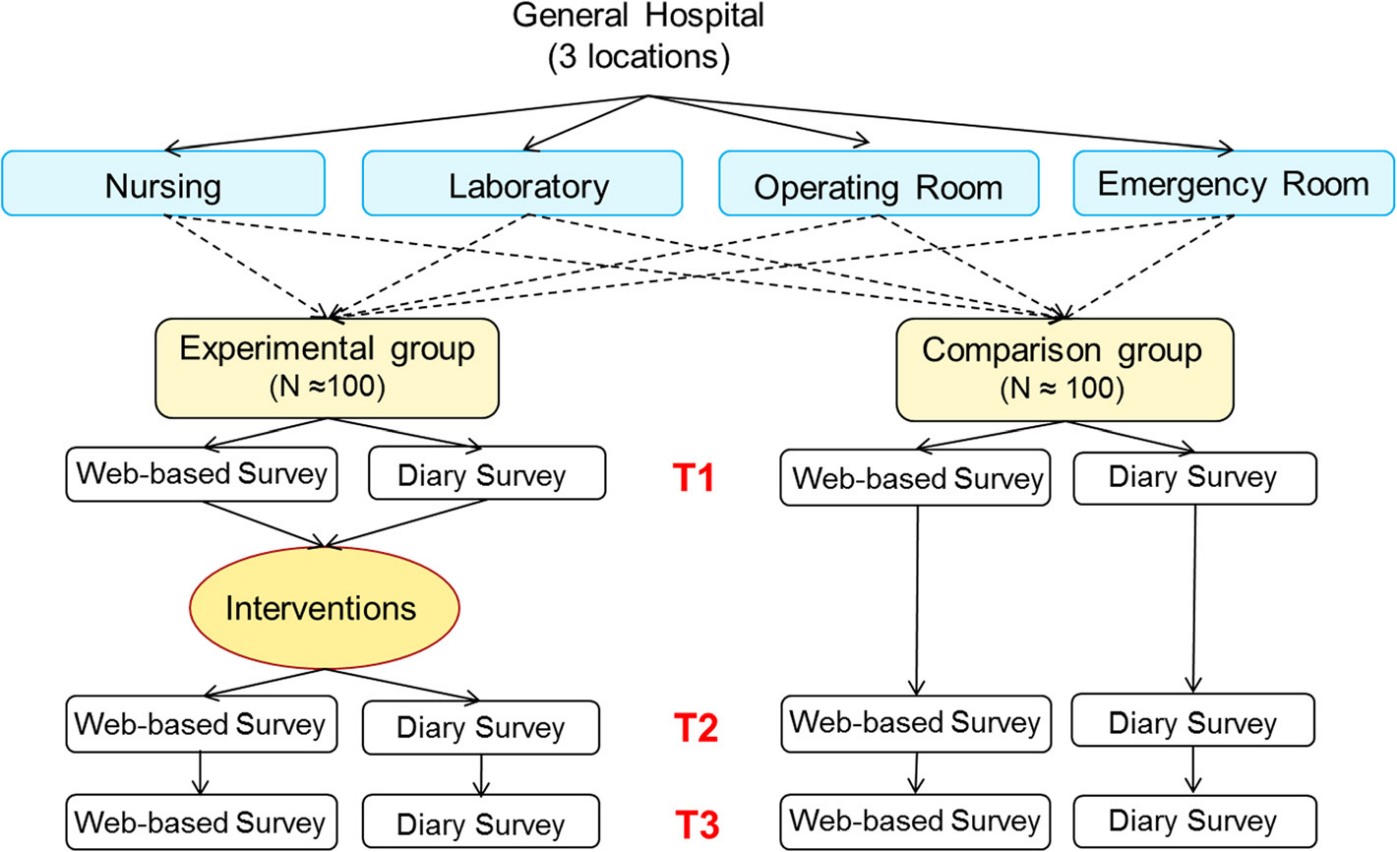
Flow chart design and measurements of the intervention study.

A quasi-experimental field study with a ‘non-equivalent control group pretest-posttest design’ will be conducted in a top general hospital with three locations in the Eastern part of Netherlands. Four existing organizational departments (consisting of a nursing department, a laboratory, an operating room department, and an emergency room department) within three locations of the hospital are chosen. All departments will provide both an intervention and a comparison group. In other words, four units become intervention groups (*n* ≈ 100) and another four become comparison groups (*n* ≈ 100). The study will comprise several successive phases, based on former experiences of the researchers with this kind of research [e.g. [20]. Two types of research can be distinguished accordingly: (1) a longitudinal web-based survey study, and (2) a longitudinal daily diary study. After the base-line measures (T1) of both studies, existing and yet to be developed interventions will be implemented within the experimental groups. Figure 1 presents a flowchart of the design and measurements. To analyze short-term and long-term effects of the interventions, follow-up measurements will be taken one (T2) and two (T3) years after the base-line measures. In addition to the follow-up measurements, a process evaluation and a cost-effectiveness analysis will be carried out.

### Participants

All employees working at the four hospital departments will be eligible to participate in the study. To obtain as much information as possible about each unit, temporary staff and apprentices will be included as well. The total group of participants will mainly consist of nurses, laboratory workers, doctors, and operating room teams, but will also include other job positions, such as management and administrative staff.

The distribution of the department units in experimental and comparison groups will be made after the baseline data analysis, in close consultation with the hospital management. Each department will provide one experimental unit. The assignment of units to the experimental group will primarily be based on their scores on the key DISC elements (i.e., are job demands, job resources, and recovery at/after work out of balance?). Furthermore, response rate, unit size and other specific unit characteristics (e.g., representativeness, planned organizational changes) will be taken into consideration. Various department units will be eligible as comparison groups. After the experimental units have been chosen, one or more of the remaining units will be selected to function as comparison unit for each experimental unit, preferably based on similarities in the work contents and target population.

From the total pool of participants in the web-based survey study, a relatively smaller group will be selected to participate in the daily diary study. Although random selection is preferred, eligibility criteria based upon specific individual information of participants hamper this approach. First of all, participants will be asked to participate in all three daily diary studies (i.e., baseline and two follow-ups), requiring reasonable prospects of keeping the same job position for at least two years. Second, to exclude employees who are still in their familiarization period, participants should be active in their current job position for at least three months at the start of the base-line measures. Third, participants should work a certain amount of hours (e.g., 20 hours) within the course of the data collection, so a balanced amount of data from both working and non-working days can be collected. Therefore, the heads of all participating units will be asked to recruit employees that meet the criteria. Every unit will provide a certain number of participants in proportion to the unit size, together making a group of 80 participants.

### Procedure

At the base-line measure, every participant will receive a unique link to the web-based survey. An electronic survey tool will randomly assign an identification number to each unique link and a daily diary survey tool will assign identification numbers to each unique device. The device numbers will be linked to the participants in a separate data file. The identification numbers of both studies will be retained and used for the follow-up measures. They are only available for the researchers and will only be used for analysis purposes. Monetary incentives will be offered to participants completing the web-based survey, as well as to participants completing the daily diary study.

The participants of the daily diary study will receive an iPod Touch® for ten consecutive days. They are asked to fill out a brief version of the internet survey on the device on two to three moments a day, on both working and non-working days. It will be investigated how people recover during and after a working day, how this influences sleep duration, sleep quality, and general health, how it influences the use of particular resources and recovery opportunities, and how emotions relate to recovery. Because three daily survey studies will be conducted (one before and two after the interventions), we are also able to investigate the influence of the interventions on a daily level, given the measured constructs.

### Measures

The measures that are used in both the baseline web-based survey and the daily diary survey are described below. With minor adjustments, the items of the web-based survey are made suitable for daily diary research (e.g., from “I need to display high levels of concentration and precision at work” to “Today I needed to display high levels of concentration and precision at work”). All adjusted items are scored on a 5-point Likert scale, ranging from 1 *(strongly disagree)* to 5 *(strongly agree)*.

The results of the interventions will be determined with the same measures. Beside the survey measures, the effectiveness of the interventions will be evaluated with more objective indicators provided by the hospital (such as company-registered workplace absenteeism, turnover rates, error and near misses indicators, and financial performance), provided that involved parties formally allow the use of this information. To control for differences between the experimental and the comparison group as well as for possible confounders, several socio-demographic variables and variables regarding location and unit will be recorded, too. Whenever possible, supervisor- and/or peer-reports will be used to check for either self-report bias in several variables or convergent evidence between different kinds of assessments (e.g., for creativity, sickness absence, or performance ratings).

### Predictor measures

*Cognitive, emotional, and physical job demands and job resources* will be measured with a well-validated version of the DISC Questionnaire (DISQ), which was particularly developed for testing the DISC Model in several languages [e.g. [10]. Example items of cognitive, emotional, and physical job demands are respectively “I need to display high levels of concentration and precision at work”, “I have to deal with people (e.g., clients, colleagues or supervisors) whose problems touch me emotionally” and “I have to lift or move heavy persons or objects (more than 10 kg)”. Example items of cognitive, emotional, and physical job resources are respectively “I have the opportunity to take a break when tasks require a lot of concentration”, “Other people (e.g., clients, colleagues or supervisors) offer me a listening ear when I have faced a threatening situation”, and “I am able to use adequate technical equipment to accomplish physically strenuous tasks”. All scales consist of three items, except for the cognitive job resources scales, which has one item extra due to psychometric properties. All items will be scored on a 5-point frequency scale, ranging from 1 *(never or very rarely)* to 5 *(very often or always)*. In the diary study, a selection of two items from each scale will be used.

*Cognitive, emotional, and physical home demands and home resources* will be measured with one item each, due to space limitations. However, when the construct of interest is relatively narrow or is unambiguous to respondents, a single-item measure may be more appropriate [21]. The items are specially developed for this study by adapting DISQ items to the private situation, e.g., “In my private situation I have to deal with a high level of physical demands” and “In my private situation I will get emotional support from others (e.g., family, friends or acquaintances) when a threatening situation occurs”. All six items will be scored on a 5-point frequency scale, ranging from 1 *(never or very rarely)* to 5 *(very often or always)*. The six items will be used in the diary study as well.

*Off-job recovery* will be measured with a scale developed by De Jonge et al. [15], which contains a cognitive, emotional, and physical component. Each component will be measured with three items, e.g., “After work, I put all thoughts of work aside” (cognitive), “After work, I emotionally distance myself from work” (emotional), “After work, I shake off the physical exertion from work” (physical). For the diary study, a selection of two items from each scale and one extra item (i.e., “I have recovered sufficiently from my last work duty”) will be used.

*Recovery at work* will be measured with three items derived from the off-job recovery scale and adapted to work breaks. Each of the three items reflects a different component (cognitive, emotional, and physical), e.g., “During a work break, I emotionally distance myself from work”. All recovery items will be scored on a 5-point frequency scale, ranging from 1 *(never or very rarely)* to 5 *(very often or always)*. In the diary study, one item will be used to measure recovery at work (i.e., “During my work break(s), I was able to recover sufficiently from my work”), and an additional item is used to measure work break duration, with possible answers ranging from “less than 15 minutes” to “more than 60 minutes”.

### Health measures

Variables in this study that will be included to measure employee health are concentration problems, emotional exhaustion, depression, physical complaints, sleep quality, and sickness absenteeism.

*Concentration problems* will be measured with four items derived from a semantic differential scale developed by Meijman [22]. The 5-point response scale has two extremes, for example “No concentration difficulties” and “Concentration difficulties”. Three items will be used in the diary study.

*Emotional exhaustion* will be measured with the well-validated Dutch version [23] of the Maslach Burnout Inventory [24]. The scale contains five items with a 7-point response scale ranging from 0 *(never)* to 6 *(always, daily)*. An example item is: “I feel emotionally drained from my work”. In the diary study, three items will be used.

*Depression* will be measured with two items from the Primary Care Evaluation of Mental Disorders patient questionnaire [25], i.e., “During the past month, have you often been bothered by feeling down, depressed, or hopeless?” and “During the past month, have you often been bothered by little interest or pleasure in doing things?”. The possible responses are 1 *(no)*, 2 *(sometimes)*, and 3 *(yes)*. The combination of these two items has been suggested to be a useful measure to detect depression in primary care [26].

*Physical complaints* refer to neck, shoulder, back, and limb problems in the last six months and will be measured with four items derived from a scale developed by Hildebrandt and Douwes [27]. The possible responses are 1 *(no)*, 2 *(sometimes)*, and 3 *(yes)*. Three items will also be used in the diary study.

*Sleep quality* will be measured by three items derived from the Maastricht Questionnaire [28], e.g., “Do you often have problems falling asleep?”. The possible responses are 1 *(no)*, 2 *(sometimes)*, and 3 *(yes)*. In the diary study, one item will be used to measure sleep quality (i.e., “How do you rate the quality of your sleep?”), with a semantic scale ranging from “very bad” to “very good”. S*leep duration* will also be assessed in the diary study, using one item (i.e., “How many hours did you sleep?”), and a scale ranging from “less than 5 hours” to “more than 9 hours”.

*Sickness absenteeism* will be measured both subjectively and objectively. Two open questions from the Dutch National Working Conditions Survey [29] will be used to measure self-reported frequency and duration of sickness absenteeism, e.g., “How many times have you been on sick leave within the last 12 months?”. Besides the self-report measures, sickness absence registrations will be provided by the Human Resources Department.

### Well-being measures

Variables in this study that will be included to measure employee well-being are job satisfaction, work motivation, and emotions.

*Job satisfaction and work motivation* will be measured by items developed by De Jonge [30]. *Job satisfaction* can be considered as unidimensional and general construct, resulting from positive and negative work experiences. It will be measured with one item, i.e., “I am satisfied with my present job”. *Work motivation* is the extent to which the work is stimulating, interesting, and challenging and will be measured with two items, e.g., “My work is meaningful”. All three items will be scored on a 5-point Likert scale, ranging from 1 *(strongly disagree)* to 5 *(strongly agree)*. The same three items will be used in the diary study.

*Emotions* will be measured only in the diary study, by using eight items of the Job Related Affective Well-Being Scale [31], e.g., “Today during work, I felt enthusiastic”. The items will be scored on a 5-point Likert scale, ranging from 1 *(strongly disagree)* to 5 *(strongly agree)*.

### Performance measures

To measure employee performance, the variables job performance, active learning, employee creativity, and counterproductive work performance will be included.

*Job performance* will be measured by six items from a scale developed by Roe, Zinovieva, Dienes and Ten Horn [32], e.g., “Compared to the standards I usually get good results from my work”. The items will be scored on a 5-point Likert scale, ranging from 1 *(strongly disagree)* to 5 *(strongly agree)*.

*Active learning* refers to the degree to which employees are enabled and stimulated to acquire new knowledge and skills, and to solve problems at their job. This scale [33] consists of four items that are scored on a 4-point frequency scale, ranging from 1 *((almost) never)* to 4 *((nearly) always)*. For example, “At work, I am challenged by new problems”.

*Employee creativity* can be defined as the generation of new and useful ideas by employees. This work-related construct is assessed by a 7-item scale, originally developed by George and Zhou [34], and translated in a well-validated Dutch version [e.g. [2]. The scale will be scored on a 5-point frequency scale ranging from 1 *(never)* to 5 *(always)*. For example, “At work I come up with new and practical ideas to improve performance”. Three items will be used for the diary study.

*Counterproductive work performance* will be measured with a selection of five items of deviant workplace behaviors from the scale developed by Kelloway, Loughlin, Barlin, and Nault [35]. Respondents will be asked to report how often they have engaged in each of the five listed behaviors in the recent past, with a 5-point frequency scale ranging from 1 *(never)* to 5 *(always)*. For instance, “intentionally worked slowly” and “blamed your coworkers for your mistakes”.

### Control measures

Next to socio-demographic characteristics (i.e., age, gender, marital status, number of children living at home, education, job position, type of work shifts, contractual working hours, actual working hours), several personal characteristic measures (i.e., overcommitment, general self-efficacy, self-oriented perfectionism) will be included to control for individual differences. Past studies have shown that each of these personal characteristics could have an influence on health, well-being, and performance-related outcomes [36-38].

*Overcommitment* reflects a respondent’s (in)ability to withdraw from work obligations and develop a more distant attitude towards job requirements and is measured with three items from the Overcommitment Scale [39]. For example, “People close to me say I sacrifice myself too much for my job”. The items will be scored on a four-point Likert scale ranging from 1 *(strongly disagree)* to 4 *(strongly agree)*.

*General self-efficacy* refers to one’s belief in one’s capability of meeting task demands in a broad array of contexts, and will be measured with the New General Self-Efficacy Scale [40]. The scale consists of eight items (e.g., “I am confident that I can perform effectively on many different tasks”), that will be scored on a 5-point Likert scale ranging from 1 *(strongly disagree)* to 5 *(strongly agree)*.

*Self-oriented perfectionism* refers to unrealistic standards and perfectionistic motivation for the self and will be measured with three items from the 15-item subscale from the Multidimensional Perfectionism Scale [41]. For instance, “I strive to be as perfect as I can be.” The items will be scored on a 7-point Likert scale, ranging from 1 *(strongly disagree)* to 7 *(strongly agree)*.

### The *DISCovery* method: risk assessment, intervention development and implementation

A participatory action approach for diagnosis, development, implementation, and evaluation of workplace interventions will be used, the so-called *DISCovery* method [42]. This method is aimed at optimizing a balance between job demands, job resources, and recovery from work. The purpose is to get insight into employee health, well-being, and performance, to investigate hindering and stimulating factors which are associated with these outcomes, and to implement workplace interventions to increase these outcomes. The *DISCovery* method consists of three successive steps: (1) a psychosocial risk diagnosis, merely based on a web-based survey and/or digital daily surveys using the DISC Model as a theoretical framework; (2) participatory action research (PAR) approach in which both employees and management are responsible for the initialization and development of interventions [43]; and (3) a work-oriented intervention program, including a process evaluation. The application of the three steps of the method in this study is outlined below.

**Figure 2:**
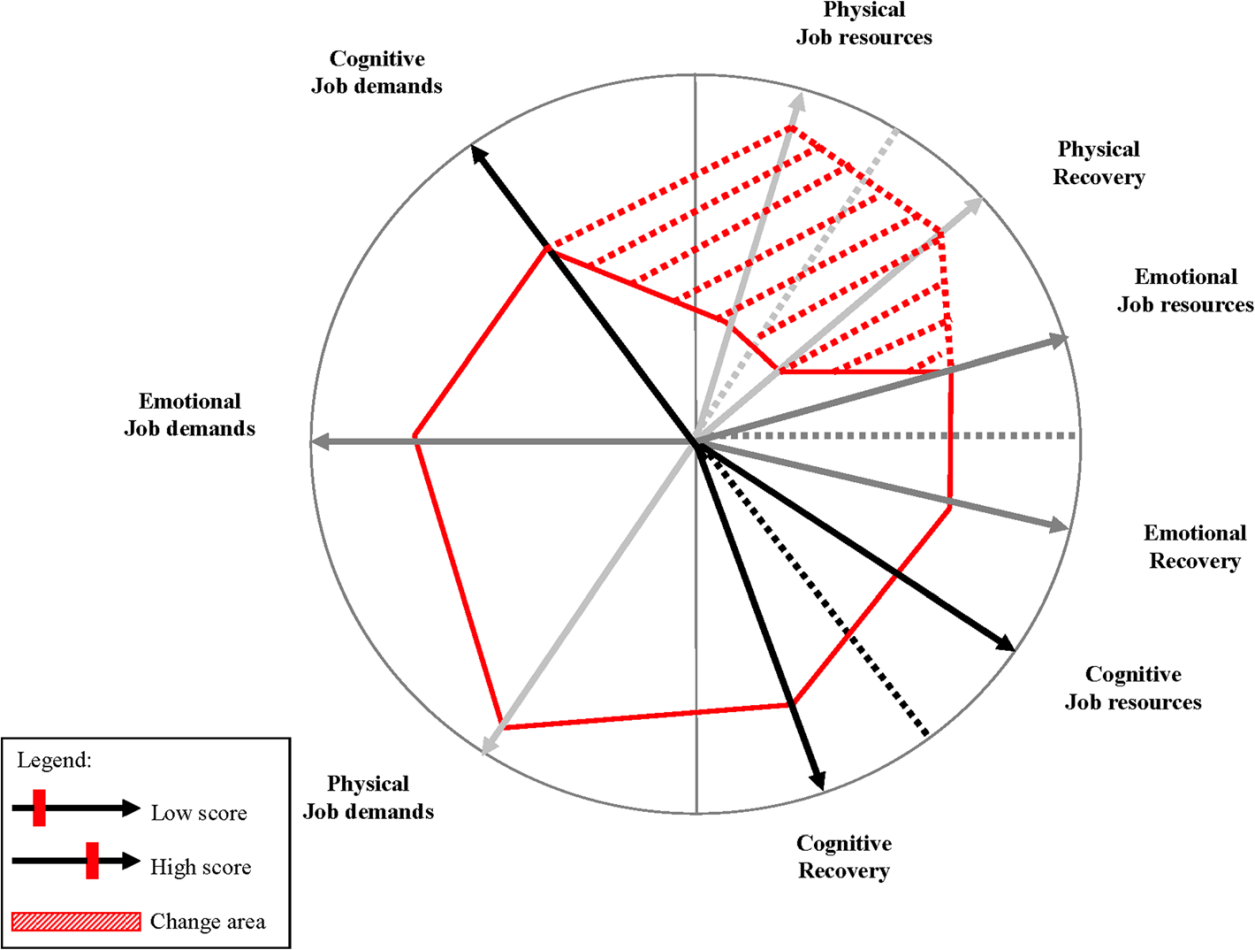
Example of a DISC risk unit-profile.

In the first step of the *DISCovery* method (i.e., the psychosocial risk diagnosis), so-called DISC risk profiles are developed for each participating unit based on baseline survey results. These profiles portray a balance between job demands, job resources, and recovery after work, and are complemented by identical profiles applied on the private situation (i.e., home demands, resources, and recovery). The latter type of profiles will function as a way to check if a lack of balance could also be explained by non-work related factors. Figure 2 shows an example of a unit-profile where the physical DISC job components seem out-of-balance (indicated by the dotted area). The DISC risk profiles will be the starting-point to generate ideas for workplace interventions.

In the second step of the *DISCovery* method, a participatory action research (PAR) approach will be used, in which both employees and management are responsible for the initialization and development of work-oriented interventions. The philosophy behind PAR is that organizational interventions designed to promote employee health cannot take place without the participation and experience of the subjects under study [44]. As a matter of fact, all people involved will become the ‘owners’ of the problems. The effectiveness of PAR in intervention research has been demonstrated empirically [e.g. [45]. Dollard and colleagues [43] pointed out that PAR as a philosophy and method embodies core ingredients of successful stress management interventions, and therefore holds promise for the reduction of stress hazards in contemporary working life. They also argued that PAR has the added potential of contributing to organizational sustainability, as organizations learn to continuously solve problems as new issues emerge. In the current study, PAR will consist of six different steps. First, feedback meetings about the results of the diagnosis with a steering group (higher management and researchers) and a project group (line management, human resources advisors, and researchers) will take place. During these meetings, preliminary ideas about interventions can already be introduced and discussed by all parties. The steering group can also veto interventions beforehand, if they seem unfeasible for any reason (e.g., hiring more staff). Second, feedback meetings are organized with each experimental unit about the results as presented in the DISC risk profile (e.g., see Figure 2). Third, subsequent to the feedback meetings, brainstorm sessions will be held with each experimental unit about possible work-oriented interventions. During these sessions an efficacious prioritization method to choose interventions will be used [42]. Every participant will receive three post-it notes to write down ideas for possible interventions that may contribute to a (partial) solution of the identified problems. This can be done either individually or in small groups of 2–3 persons. Next, the post-it notes with ideas for interventions will be pasted on a flip-chart. Ideas are clarified to all participants and grouped together in different intervention types. A list of interventions will be written down, including possible ideas of the project and steering group. All participants receive three stickers, which they paste on the flip-chart behind their individually preferred intervention. They can either make a personal top-3 or put all three stickers behind one particular intervention. This will result in a specific ranking list with a top-3 of interventions for every experimental unit. Fourth, the steering and project group will be consulted about the several top-3 intervention lists and possible actions to be taken to implement the interventions. Most important are urgency, feasibility, and individual, departmental and organizational values, reflected in short and long term actions. Also, employee preferences should weigh heavily in the final choice of interventions. Fifth, conclusions of the fourth step will be reported to the experimental units and they will be asked for reactions and commitment. Sixth, higher management makes a final decision in consultation with employees, lower management and researchers, about which interventions will be implemented on each experimental unit.

In the third step of the *DISCovery* method, workplace interventions will be actually implemented, some of which already exist and others that are to be developed. During the process of developing and implementing interventions, the researchers will be supported by external consultants wherever necessary. The interventions are primarily work-oriented rather than worker-oriented, in order to provide more effective and sustainable solutions [17]. They will mainly be targeted at matching cognitive, emotional, or physical aspects of work and/or recovery, depending on the specific unit-profiles. For instance, if a unit-profile displays that (high) physical job demands, (low) physical job resources and (low) physical recovery are out-of-balance, an intervention aimed at increasing physical job resources or improving physical recovery at/after work can be chosen during the PAR-method in order to counteract the relatively high physical job demands. One intervention possibility is to check if there is sufficient adequate technical equipment to accomplish physically strenuous tasks. It can also be important to find out if already available physical resources are used correctly by the employees [8]. Another example is the introduction of smarter rosters directed at limiting long working hours and night work [46]. Interventions could also be implemented by means of a workshop, for example a workshop ‘how to cope with physical demands by effective physical recovery’ [42]. To conclude, based on the outcome of the PAR-method, the precise intervention program will be determined.

### Intervention evaluation

We will evaluate the short- and long-term effects of the workplace interventions with the first and second follow-up surveys, respectively. After the first follow-up survey, we will investigate if the interventions have led to higher motivation, improved performance and better health. After the second follow-up survey it can be determined if the expected positive effects of the interventions were also noticeable one year later. The results of the follow-up surveys will be displayed in the DISC risk profile for each unit, next to the base-line scores. As such, the change over time within specific job demands, job resources, and recovery opportunities will be made visible for all parties involved.

With an econometric cost-effectiveness analysis [47] carried out by an econometrician, intervention costs will be compared with the obtained benefits due to reduced sickness absence, reduced turnover, improved work productivity, reduced number of incidents, and increased economic performance. Dividing the difference in total costs between the intervention and comparison groups (ΔC) by the difference in effects (ΔE), will result in incremental cost-effectiveness ratios (ICERs) in terms of health, well-being, and performance-related outcomes. The time horizon for the cost-effectiveness analysis will be 18 months, starting at the kick-off of the interventions.

Since the study takes place in a dynamic environment, a wide area of external factors could influence the results. A process evaluation will be carried out to gain insight into factors that either stimulated or hindered successful implementation, as well as the effectiveness of the interventions [48]. First, the heads of the participating hospital units will be asked to keep track of all important changes and events on and surrounding their unit in a logbook (e.g., reorganization, interpersonal conflicts, new equipment). They will receive a periodical reminder to fill out the logbook, from the beginning of the base-line measures until the end of the follow-up measures. The logbook will be used to interpret possible changes in performance, well-being, and health in both the intervention and the comparison groups. If necessary, the information in the logbooks will be extended by interviewing the heads of department and other staff members. Second, a semi-structured questionnaire will be used for all participating groups to count how many and what kind of actions were taken as part of the intervention. In this way, it can be examined if the comparison groups implemented interventions on their own initiative. Finally, cultural differences between the locations and units that either have a positive or a negative influence on the effectiveness of the interventions will be mapped. Overall, intervention evaluation criteria proposed by Scharf et al. [49] will be followed as much as possible, such as participation of workers and management and the inclusion of different organizational levels.

### Statistical analysis

Hierarchical regression analysis (SPSS) and structural equation modeling (LISREL or AMOS) will be applied to test cross-sectional baseline relations between specific types of job demands, job resources, recovery, and job-related outcomes. In order to analyze causal associations within the three different waves of all digital surveys, structural equation modeling will be used, as this technique is more useful to rule out alternative assumptions. Multilevel regression analysis (MLwiN) will be used to investigate the relation between job demands, job resources, recovery, and health/performance outcomes, based on data from the three daily surveys (level 1: three waves; level 2: day-level predictor and control variables; level 3: person-level control variables). To evaluate the results of the interventions after the follow-up measures, multilevel repeated measures analysis will be performed using MLwiN. This technique has several advantages compared to repeated measures MANOVA, such as the inclusion of cases with incomplete data and less restrictive missing data assumptions. Finally, to study change in organizational interventions, knowledge about the type of change underlying the instruments used is needed. Next to assessing baseline factorial validity and reliability, the factorial stability over time (known as alpha-beta-gamma change) of the key measures will be examined [50]. Drop-outs will be documented and included in the data-analysis to the point of drop-out. Possible attrition effects (e.g., spurious and under- or overestimated relationships among the study variables) will be analyzed according to the guidelines by Goodman and Blum [51].

### Sample size calculation

Sample size calculation is based on emotional exhaustion, measured by the Dutch version [23] of the Maslach Burnout Inventory [24]. This measure is chosen because of the availability of norm scores for nurses, which is a frequently occurring job position in both the intervention and comparison groups. The score ranges between 1 and 7, with an average score of M = 1.62 and a standard deviation of SD = .85. Setting alpha at 0.05, beta as 0.20 and Δ = .43 (half a standard deviation as a clinically minimal relevant difference [52]), results in a required N = 148 (N = 74 for the intervention group and N = 74 for the comparison group) [53]. However, the total number of employees in the four participating departments (N ≈ 200) somewhat exceeds the required sample size. Taking drop-outs into account, this sample is expected to be large enough to detect significant effects.

### Ethical considerations

The Medical Ethics Committee Region Arnhem-Nijmegen of the UMC St. Radboud has exempted the current study from ethical approval: the committee confirmed that the current study is carried out in the Netherlands in accordance with the applicable rules concerning the review of research ethics committees and informed consent (reference number: 2012/546). In addition, both higher and lower management of the hospital have given their consent after ample presentation of the research plan. Finally, potential participants have been informed about the research plan, the nature of the study and voluntary participation, by means of an introduction letter and information gatherings at every participating unit. Participants in the daily diary studies will be asked to sign an informed consent. Throughout the whole research project, it will be stressed that employees participate on a voluntary basis, that confidentiality is guaranteed, and that they can withdraw from the study at any moment.

## Discussion

Health care workers in today’s general hospitals have to deal with high levels of job demands, which could have negative effects on their health, well-being, and job performance. Prior research has indicated that job resources and recovery opportunities can counteract these negative effects and improve positive work-related outcomes (e.g., creativity and active learning behavior), specifically if they match with the type of job demands (i.e., cognitive, emotional, or physical). So far, the translation from theory into practice has not yet been fully made: it is still unclear how the balance between job demands, job resources, and recovery opportunities can be optimized by means of workplace interventions. The current research will contribute to filling this gap between theory and practice. The aim of the DISCovery project is to develop and implement tailored work-oriented interventions, to improve a healthy working life and job performance in health care.

### Strengths and limitations of the DISCovery project

Because a systematic and theory-driven analysis of work-related risk factors is often lacking in stress intervention research [48,54], a first strength of the study is the theory-driven diagnosis of specific risk factors with both a longitudinal survey study and a longitudinal daily diary study. A second strength is the use of the Participatory Action Research (PAR) approach, which involves multiple stakeholders and allows health care employees to participate in the development and implementation of the interventions. This approach will stimulate problem ownership and commitment at all levels of the organization and has the potential to contribute to organizational sustainability [43]. A third strength is that the interventions will be primarily work-oriented, targeted at the source of job stress problems. Whereas individual-level strategies can offer short-term solutions, addressing the sources of job stress (i.e., the stressful working situation) can provide more effective and sustainable solutions [17]. Furthermore, interventions taking place at the workplace, including multiple, representative health care settings (i.e., different departments in a general hospital) and a diverse, heterogeneous sample (i.e., different job positions), will provide good external validity of the findings with regard to other hospitals and health care institutions. A fourth strength is that, next to self-report measures, more objective measures such as sickness absenteeism and turnover rates will be collected. A cost-effectiveness analysis will be carried out to compare intervention costs with a number of ‘hard’ outcomes, such as sickness absenteeism and turnover rates, safety and error rates, and work productivity. In previous research, there has generally been a lack of inclusion of ‘hard’ measures next to ‘soft’ measures. A final strength of this study is that different types of interventions can be compared on respectively similar outcomes, which is an important contribution to both theory and practice [54].

Besides strengths, a few limitations can be identified. One limitation of the study is that the design is not truly experimental. For practical and ethical reasons it is impossible to randomly assign participants to the intervention and comparison groups. The participating units in this project are existing organizational units and, in line with the participative nature of the research, the hospital management will have an important vote in the distribution of the units into intervention and comparison groups. However, various units from each of the four departments will be eligible as comparison groups. This provides the opportunity to compare different units, and to make an adequate selection of a comparison group for each intervention unit, based on similarities in work content and target population (e.g., job position, sex, age, educational level). Another limitation is that a wide area of external factors could influence the results (e.g., reorganization, company and/or departmental policy changes), since the study takes place in a dynamic environment. Yet, a process evaluation will give insights into the kind and the extent of external influences.

In spite of these limitations, the DISCovery project offers a carefully considered triangulation of research methodologies to develop and implement tailored work-oriented interventions and to assess the effects on health, well-being, and performance-related outcomes. The project is currently in progress. The dissemination of results is planned for 2014.

## Competing interests

The authors declare that they have no competing interests.

## Authors’ contributions

IMWN, JDJ, JMPG, and ILDH were involved in the design of the study. IMWN drafted the manuscript and performed the statistical analysis. JDJ, JMPG, and ILDH critically reviewed the manuscript. All authors read and approved the final manuscript.

## Pre-publication history

The pre-publication history for this paper can be accessed here:

http://www.biomedcentral.com/1472-6963/13/66/prepub
